# Transcriptome sequencing of microglial cells stimulated with TLR3 and TLR4 ligands

**DOI:** 10.1186/s12864-015-1728-5

**Published:** 2015-07-10

**Authors:** Amitabh Das, Jin Choul Chai, Sun Hwa Kim, Young Seek Lee, Kyoung Sun Park, Kyoung Hwa Jung, Young Gyu Chai

**Affiliations:** Department of Bionanotechnology, Hanyang University, Seoul, 133-791 Republic of Korea; Department of Molecular & Life Sciences, Hanyang University, Ansan, 426-791 Republic of Korea; Institute of Natural Science & Technology, Hanyang University, Ansan, 426-791 South Korea

**Keywords:** Gene regulation, Innate immunity, Toll-like receptor, Microglia, RNA sequencing

## Abstract

**Background:**

Resident macrophages in the CNS microglia become activated and produce proinflammatory molecules upon encountering bacteria or viruses. TLRs are a phylogenetically conserved diverse family of sensors that drive innate immune responses following interactions with PAMPs. TLR3 and TLR4 recognize viral dsRNA Poly (I:C) and bacterial endotoxin LPS, respectively. Importantly, these receptors differ in their downstream adaptor molecules. Thus far, only a few studies have investigated the effects of TLR3 and TLR4 in macrophages. However, a genome-wide search for the effects of these TLRs has not been performed in microglia using RNA-seq. Gene expression patterns were determined for the BV-2 microglial cell line when stimulated with viral dsRNA Poly (I:C) or bacterial endotoxin LPS to identify novel transcribed genes, as well as investigate how differences in downstream signaling could influence gene expression in innate immunity.

**Results:**

Sequencing assessment and quality evaluation revealed that common and unique patterns of proinflammatory genes were significantly up-regulated in response to TLR3 and TLR4 stimulation. However, the IFN/viral response gene showed a stronger response to TLR3 stimulation than to TLR4 stimulation. Unexpectedly, TLR3 and TLR4 stimulation did not activate IFN-ß and IRF3 in BV-2 microglia. Most importantly, we observed that previously unidentified transcription factors (TFs) (i.e., IRF1, IRF7, and IRF9) and the epigenetic regulators KDM4A and DNMT3L were significantly up-regulated in both TLR3- and TLR4-stimulated microglia. We also identified 29 previously unidentified genes that are important in immune regulation. In addition, we confirmed the expressions of key inflammatory genes as well as pro-inflammatory mediators in the supernatants were significantly induced in TLR3-and TLR4-stimulated primary microglial cells. Moreover, transcriptional start sites (TSSs) and isoforms, as well as differential promoter usage, revealed a complex pattern of transcriptional and post-transcriptional gene regulation upon infection with TLR3 and TLR4. Furthermore, TF motif analysis (-950 to +50 bp of the 5′ upstream promoters) revealed that the DNA sequences for NF-κB, IRF1, and STAT1 were significantly enriched in TLR3- and TLR4-stimulated microglia.

**Conclusions:**

These unprecedented findings not only permit a comparison of TLR3-and TLR4-stimulated genes but also identify new genes that have not been previously implicated in innate immunity.

**Electronic supplementary material:**

The online version of this article (doi:10.1186/s12864-015-1728-5) contains supplementary material, which is available to authorized users.

## Background

The immune system includes cells that can recognize Toll-like receptors (TLRs) and respond to TLR ligands by expressing several genes that are implicated in innate immunity, which is the first line of mammalian defense against invading pathogens. TLRs distinguish conserved patterns that are derived from microbial pathogens, which are recognized as pathogen-associated molecular patterns (PAMPs), including TLR3 (viral double-stranded RNA (dsRNA)), TLR7/8 (single-stranded RNA (ssRNA)) and TLR9 (DNA) [1]. The interaction of TLRs with a PAMP, such as bacterial endotoxin lipopolysaccharide (LPS) and viral dsRNA, triggers several signaling cascades that lead to the induction of numerous target genes involved in inflammation [2]. Polyinosinic-polycytidylic acid Poly (I:C) is a synthetic analog of viral dsRNA, which is a PAMP that is generated during the replication of RNA and DNA viruses and is recognized by TLR3, while TLR4 is the receptor that recognizes LPS [2]. TLR3 and TLR4 are unique in that these receptors can transmit signals through the myeloid differentiation factor 88 (MyD88)-independent, TIR-domain–containing adaptor inducing interferon ß (TRIF)-dependent pathway [3]. Next, MyD88 and TRIF induce the key transcription factor (TF) nuclear factor κB (NF-κB) signalosome and mitogen-activated protein kinases (MAPKs); both pathways facilitate the transcription and stabilization of mRNAs for proinflammatory mediators, including cyclooxygenase-2 (COX-2), interleukin-6 (IL-6), IL-1ß, tumor necrosis factor-α (TNF-α), and IL-12 [4]. Although these signaling molecules and their cascades have been explored in macrophages, the dynamic outcome of genome-wide approaches and the effects of TLR3 vs TLR4 in murine microglia remain poorly understood.

Microglia, which are a type of glial cell, are resident macrophages of the brain and spinal cord that act as primary effector cells and these cells play an important role in the brain’s innate immunity, neuronal homeostasis, and neuroinflammatory pathologies [5]. Microglial cells become rapidly activated in response to infection, inflammation or injury, and their activation is associated with the production and secretion of reactive oxygen species (ROS), nitric oxide (NO) and prostaglandin E2 (PGE2), and a variety of proinflammatory cytokines, including IL-1ß, IL-6 and TNF-α [6]. Although microglial activation is considered a protective mechanism involved in the clearance of pathogen infection and in the regulation of tissue repair and recovery, excessive or persistent activation as an uncontrolled immune response stimulates and increases the production of neurotoxic pro-inflammatory mediators, causing neuroinflammation and neurological disorders including Alzheimer’s disease (AD), Parkinson’s disease (PD), brain ischemia and multiple sclerosis (MS) [6]. Currently, the heterogeneity of microglia in terms of their activation phenotypes and their role in neurodegenerative disease progression and repair is an area of active investigation. Microglia express all known TLRs to detect and respond robustly to both Poly (I:C) and LPS [7]. Previous studies have demonstrated that LPS stimulation induces the gene expression of IL-1ß, IL-6, TNF-α, inducible nitric oxide synthase (iNOS) and prostaglandin-endoperoxide synthase 2 (PTGS-2), as well as the production of NO and PGE2 in microglial cell cultures [6]. In contrast, Poly (I:C) activates antiviral and inflammatory genes independent of the adaptor protein MyD88, which is required for all other TLRs [3]. However, the full effect on gene up-regulation and the basis of this synergy is unknown.

Although a few studies have compared the effects of Poly (I:C) vs LPS on murine microglia or macrophages [8, 9], thus far, a genome-wide search for the effects of Poly (I:C) vs LPS using the RNA-seq specifically has not been performed in murine microglia. Therefore, we performed gene array studies and comparative gene expression profiling analyses of BV-2 cells treated with Poly (I:C) and LPS using RNA-seq. In this study we examined BV-2 cell lines as a model of inflammation studies. This is one of the major uses of microglia. Previously, others reports demonstrated that BV-2 cell lines have close resemblance to primary brain microglia [10–12]. Since BV-2 cells are easy to culture, they are an important tool to study not only inflammatory processes [12], but also phagocytosis [13]. Increasing yield and homogeneity of cells *in vitro* culture allows for enlarged data output compared to most *in vivo* experiments. To the best of our knowledge, this study is the first to apply these approaches to assess the differences in response induced by different TLR ligands and their consequences with respect to global gene expression in BV-2 microglial cells.

## Methods

### Cell culture and stimulation

Mouse microglial BV-2 cells were grown in high-glucose Dulbecco’s modified Eagle’s medium (DMEM) supplemented with 10 % fetal bovine serum (FBS) (catalog # 26140), 100 IU/ml penicillin, and 10 μg/ml streptomycin (catalog # 15140; Invitrogen, USA). The cells were maintained in a humidified incubator with 95 % air and a 5 % CO_2_ atmosphere at 37 °C. The cells were incubated with LPS (10 ng/ml, Sigma-Aldrich) and Poly (I:C) (5 μg/ml, Sigma-Aldrich) for the specified times under normal culture conditions. The medium, which contained the appropriate agents, was replaced every other day. The NF-κB pathway Inhibitor BAY11-7082 was purchased from Calbiochem (San Diego, CA, USA). Unless otherwise indicated, BAY11-7082 was freshly dissolved before use in the experiments. In this study, we used 10 μM of BAY11-7082 [14–16]. Primary microglial cells were isolated from 3-day-old ICR mice as previously described [17, 18]. All experimental protocols were conducted in accordance with Institutional Animal Care and Use Committee (IACUC) guidelines and were approved by the IACUC committee at Hanyang University (HY-IACUC-2014-0164A). Briefly, whole brains of neonatal mice were taken; blood vessel and meninges were carefully removed. Then, the whole brains of 12 mice were pooled together, finely minced, and digested with Neural Tissue Dissociation Kit-Postnatal Neurons (Miltenyi Biotec-130-094-802). Next, digested cells pass through 70-μm nylon cell strainer (BD Biosciene) and were seeded in poly-L-lysine-coated T-75 flask in DMEM/nutrient mixture F-12 (DMEM/F12, 1:1) containing 20 % FBS (catalog # 26140), 100 IU/ml penicillin and 10 μg/ml streptomycin (catalog # 15140) from Invitrogen (CA, USA). The cells were maintained in a humidified incubator with a 95 % air/5 % CO_2_ atmosphere at 37 °C. The medium was changed every 2–3 days. After two weeks in culture, mixed glial cell cultures are shaken at 150 rpm at 37 °C for 45 min, and the glial cell suspension was collected from each flask and seeded on poly-L-lysine coated cell culture plate. Microglial cells were sub-plated and used for further experiments.

### Total RNA extraction

Total RNA (~8 μg) was extracted using TRIzol (Life Technologies, Carlsbad, CA, USA) according to the manufacturer’s instructions. Briefly, 200 μl of chloroform was added, and the tubes with the lysis mixture were gently inverted for 5 min. The mixture was centrifuged at 12,000 x *g* for 15 min at 4 °C, and the clear upper solution was placed into a new tube, to which 500 μl of isopropanol was added. The tubes were inverted before incubation on ice for 1 h. The lysis mixture was centrifuged at 12,000 x *g* for 10 min at 4 °C, and the isopropanol was decanted. Ice-cold 70 % ethanol was added to the RNA pellet for gentle washing. The ethanol was removed after centrifugation as indicated above for 10 min. The RNA pellets were dried at room temperature for 5–10 min before reconstitution in 20 μl of RNase-free water, and the RNA was treated with RNase-free DNase I (Promega, WI, USA). The RNA quality was assessed using an Agilent 2100 Bioanalyzer with an RNA 6000 Nano Chip (Agilent Technologies, Waldbronn, Germany), and the quantity was determined using a spectrophotometer (NanoDrop Technologies, Wilmington, DE, USA).

### Quantitative real-time RT-PCR (qRT-PCR)

The reverse transcription of the RNA samples was performed as previously described [19] using 2 μg of total RNA, 1 μl of random hexamers (per reaction) and a Prime Script 1st strand cDNA Synthesis Kit (Takara, Japan). The random hexamers and RNA templates were mixed and denatured at 65 °C for 5 min, followed by cooling for 2 min on ice. Prime Script buffer (5x), RTase and RNAse inhibitor were added to the cooled template mixture and incubated for 1 h at 50 °C before enzyme inactivation at 70 °C for 15 min. qRT-PCR was performed using SYBR Green PCR Master Mix (Takara Bio Inc., Shiga, Japan) and a 7500 Fast Real-time PCR System (Applied Biosystems, Foster City, USA). Glyceraldehyde-3-phosphate dehydrogenase (GAPDH) was used as an internal control. Complementary DNA samples were diluted 1.5-fold, and qRT-PCT was performed using an AB-7500 Real-time PCR System (Applied Biosystems, Foster City, CA, USA) with SYBR Premix Ex-Taq II (Takara Bio, Shiga, Japan) according to the manufacturer’s instructions. The reactions were performed in a total volume of 20 μl that contained 0.4 mM of each primer (Table 1). Each PCR run included a no-template control that included water instead of cDNA and a reverse transcriptase-negative control for each gene. Triplicate measurements were performed for all reactions. Different samples were evaluated using 96-well plates for gene expression experiments, and all samples were analyzed on a single plate for endogenous control determination. The results were analyzed using the critical threshold (∆C_T_) and comparative critical threshold (∆∆C_T_) methods in the AB-7500 software program with the Norm finder and geNorm-plus algorithms. The primers were designed using Primer Express software (Applied Biosystems, Foster City, CA, USA).

**Table 1:** List of primers used in qRT-PCR studies

Gene designation	Forward sequence (5′ - >3′)	Reverse sequence (5′ - >3′)
*TNF-*α	CAG GCG GTG CCT ATG TCT C	CGA TCA CCC CGA AGT TCA GTA G
*ILIß*	GAA ATG CCA CCT TTT GAC AGT G	CTG GAT GCT CTC ATC AGG ACA
*IL1A*	TCTATGATGCAAGCTATGGCTCA	CGGCTCTCCTTGAAGGTGA
*PTGS-2*	TTCCAATCCATGTCAAAACCGT	AGTCCGGGTACAGTCACACTT
*CCL-3*	TGTACCATGACACTCTGCAAC	CAACGATGAATTGGCGTGGAA
*CCL-4*	TTCCTGCTGTTTCTCTTACACCT	CTGTCTGCCTCTTTTGGTCAG
*CCL-7*	CCACATGCTGCTATGTCAAGA	ACACCGACTACTGGTGATCCT
*CXCl10*	TGC TGG GTC TGA GTG GGA CT	CCC TAT GGC CCT CAT TCT CAC
*IRF-1*	ATG CCA ATC ACT CGA ATG CG	TTG TAT CGG CCT GTG TGA ATG
*IRF-7*	GCGTACCCTGGAAGCATTTC	GCACAGCGGAAGTTGGTCT
*JUNB*	CTATCGGGGTCTCAAGGGTC	CTGTTGGGGACGATCAAGC
*NFκBIA*	AGG CTT CTG GGC CTT ATG TG	TGC TTC TCT CGC CAG GAA TAC
*CLEC4E*	TGTCGTAACATATCGCAGCTC	GGACAGCAATTCTTGACTGAACC
*GPR84*	TCTCATTGCTCTAGGACGCTAC	AGACAAAAACATTCCAGAGGGG
*SLC15A3*	GAACGCGCTGCCTTCTTTG	CAGGCTGAGCGAGATAGTGAG
*KDM4A*	GAC CAC ACT CTG CCC ACA C	TCC TGG GGT ATT TCC AGA CA
*IFN-b*	AGCTCCAAGAAAGGACGAACA	GCCCTGTAGGTGAGGTTGAT
*IRF3*	GAG AGC CGA ACG AGG TTC AG	CTT CCA GGT TGA CAC GTC CG
*KLF7*	AGT GGA CAT TTT GCT CTC TCG	GTT AAT GAG GTC ACT GCG TTG A
*IRAK3*	GTTCTACTCCTGTTCCGTCACC	GTCCCGTTGCTCATATAGGGATA
*GAPDH*	TGCGACTTCAACAGCAACTC	CTTGCTCAGTGTCCTTGCTG

### cDNA library preparation for RNA-seq

Total RNA was extracted from each group of BV-2 cells i.e., control 2 h (2 samples), control 4 h (2 samples), LPS 2 h (2 samples), LPS 4 h (2 samples), Poly (I:C) 2 h (2 samples) and Poly (I:C) 4 h (2 samples), using TRIzol® (Life Technologies) according to the manufacturer’s instructions. For RNA-Seq, RNA libraries were created from each group of BV-2 cells using a NEBNext® Ultra™ Directional RNA Library Preparation Kit from Illumina®. The first step in the workflow involved the removal of ribosomal RNA using an RNAMius™ Transcriptome Isolation Kit (Life Technologies). Following purification, total RNA was fragmented into small pieces using divalent cations at elevated temperatures. The cleaved RNA fragments were subjected to first-strand cDNA synthesis using reverse transcriptase and random primers, followed by second-strand cDNA synthesis using DNA polymerase I and RNase H. The cDNA fragments were subsequently processed using an end-repair reaction after the addition of a single ‘A’ base, followed by adapter ligation. The products of these reactions were purified and enriched through PCR to generate the final cDNA library. The cDNA fragments were sequenced using an Illumina HiSeq2500 System (101 cycles, paired-end (PE) lane) (National Instrumentation Center for Environmental Management in Seoul National University). Biological duplicate RNA sequencing was performed on twelve independent RNA samples from the treated BV-2 cells.

### Differential gene expression analysis

Raw sequence files were subjected to quality control analysis using FastQC software (http://www.bioinformatics.babraham.ac.uk/projects/fastqc/). To ensure high-quality data, we clipped the adapters and trimmed the reads using the FASTX-Toolkit (http://hannonlab.cshl.edu/fastx_toolkit/). The quality-checked reads for each condition were processed using the TopHat version 2.0.10 software package (Bowtie 2 version 2.2.1 software) as FASTQ files to analyze differentially expressed genes [20]. The reads were mapped to the reference genome [*Mus musculus* UCSC mm10 sequence], and the alignment files were generated as BAM files. These files were used as the input for Cufflinks software, which is a complementary method used to generate assembled transcripts for each condition; the abundance was evaluated using read data. The “fragments per kilobase per million map reads” (FPKM) values were calculated for each gene to normalize the data [21]. These assemblies are used with the Cuffquant and Cuffdiff tools from the Cufflinks 2.2.1 package to calculate the differential expression levels and to evaluate the statistical significance of the detected alterations [22]. RNA-seq experiments were normalized and visualized using HOMER software (http://homer.salk.edu/homer/) after preparing custom tracks for the UCSC Genome browser (http://genome.ucsc.edu/).

### Functional annotation

DAVID (Database for Annotation, Visualization and Integrated Discovery) version 6.7 software (http://david.abcc.ncifcrf.gov/home.jsp) was used to determine the most functional annotation of significant genes in the datasets as previously described [23]. The DAVID program calculates a modified Fisher’s exact *P* value to demonstrate gene ontology (GO) or molecular pathway enrichment. *P* values less than 0.05 were considered strongly enriched in the annotation category.

### Canonical pathway analysis of datasets

Ingenuity Pathway Analysis (IPA) (Ingenuity Systems, http://www.ingenuity.com, Mountain View, CA, USA) was conducted to analyze the most significant canonical pathways in datasets as previously described [19, 24]. The genes from datasets associated with canonical pathways in the Ingenuity Pathways Knowledge Base (IPAKB) were considered for literary analysis. The significance of the associations between datasets and canonical pathways was measured in the following manner: (1) the ratio of the number of genes from the dataset that mapped to a canonical pathway divided by the total number of genes that mapped to the same canonical pathway; and (2) Fisher’s exact test for a *P* value indicating the probability that the association could be explained by chance. After uploading the datasets, gene identifiers were mapped to corresponding gene objects, and the genes were overlaid onto a global molecular network in the IPAKB. Gene networks were algorithmically generated based on connectivity.

### Graphical representation of networks and pathways

The molecules from the normalized filtered RNA-seq dataset were each mapped to corresponding objects in the IPAKB for network generation. A fold change cutoff of up-regulated genes (≥1.5 log_2_-fold) was set to identify significantly and differentially regulated genes in BV-2 microglial cells at 2 and 4 h after Poly (I:C) and LPS stimulation. The graphical representation of molecular relationships between genes and gene products is based on genes or gene products, which are represented as nodes, and the biological relationship between two nodes is represented as an edge (line). All edges were supported by at least one reference from the literature, textbook or canonical information in the IPAKB. The node color intensity indicates the degree of up-regulation (red). The nodes are displayed using shapes to represent functional classes of gene products.

### Transcription factor binding motif enrichment analysis

NCBI reference sequence mRNA accession numbers were subjected to transcription factor binding motif analysis using the web-based software Pscan (http://159.149.109.9/pscan/) [25]. The JASPAR database of transcription binding factor sequences was analyzed using enriched groups of -950 base pair (bp) sequences to +50 bp of the 5′ upstream promoters [26]. The range of -950 to +50 was selected from the range options in Pscan software to obtain the best coverage for a -1000 to +50 bp range.

### Enzyme-linked immunosorbent assay (ELISA)

Primary microglial cells were cultured in the same condition as above. Primary microglial cells were treated with Poly (I:C) (5 μg/ml) and (LPS 10 ng/mL), for 2 h and 4 h. After treatment, the concentration of the pro-inflammatory mediators DNMT3L, TNF-α, IL-1ß and CCL4 were determined in cell culture supernatants using the mouse ELISA kit according to the manufacturer’s protocol. The mouse ELISA kit DNMT3L from Elabscience (Wuhan, China) and TNF-α, IL-1ß and CCL4 from Komabiotec (Seoul, Korea).

### Statistical analysis

The data were analyzed using Origin Pro 8 software (Origin Lab Corporation, Northampton, MA, USA). Each value is expressed as the mean ± standard error of the mean (SEM). The statistical analysis was performed using SPSS 17.0 software (SPSS Inc., IL, USA). The data were tested using a one-way ANOVA, followed by Tukey’s HSD post-hoc test. ^*^P <0.05 and ^**^P <0.001 were considered significant.

## Results

### RNA-Seq transcriptional profiles of murine microglia in response to TLR3 vs TLR4

**Fig. 1:**
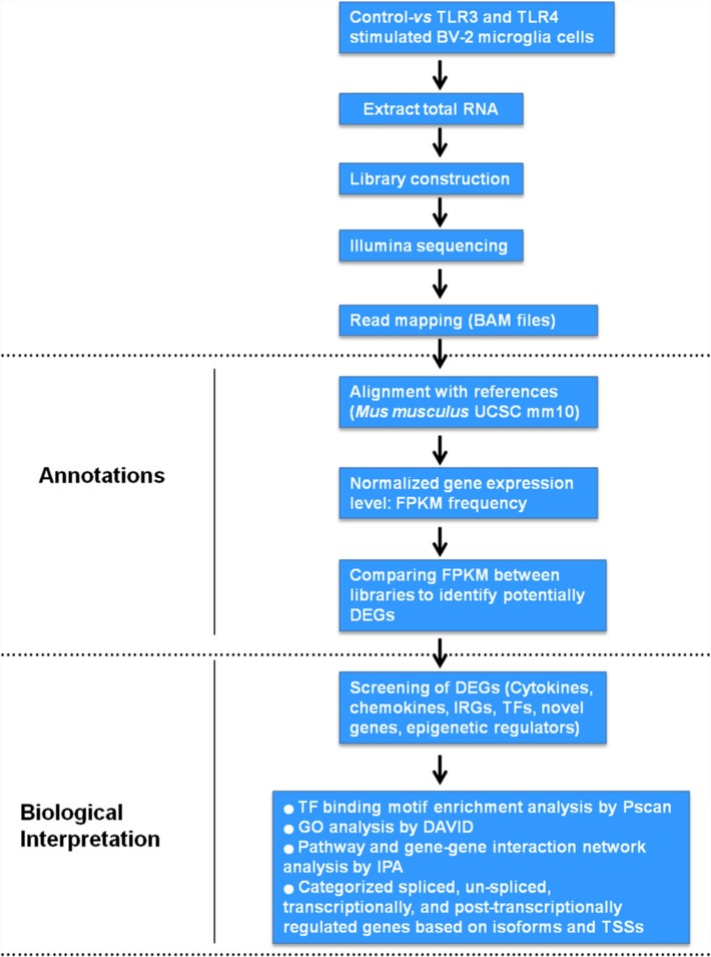
Schematic outline of experiments and data analysis

To establish a high-resolution transcriptome in response to TLR stimulation, we treated BV-2 microglial cells with Poly (I:C) or LPS for 2 and 4 h before cDNA library preparation for RNA-seq experiments. The RNA-seq transcriptional analysis was performed using two independent samples (biological replicates) of each treatment. The data from all experiments (each group) were combined, and the genes whose levels of expression significantly differed were identified. We used a 1 % false discovery rate (FDR), *P* <0.001, and fold change ≥1.5 log_2_ for up- or down-regulation as the criteria for defining differentially expressed genes. Of note, using qRT-PCR, we found that most of the inflammatory response-related genes were up-regulated at the 2 and 4 h time points (data not shown). We chose these time points for transcriptional profiling; these time points were also used in other studies [27, 28] that investigated the general induction pattern of microglial activation by Poly (I:C) or LPS. Details outline of the experiments is depicted in Fig. 1.

**Fig. 2:**
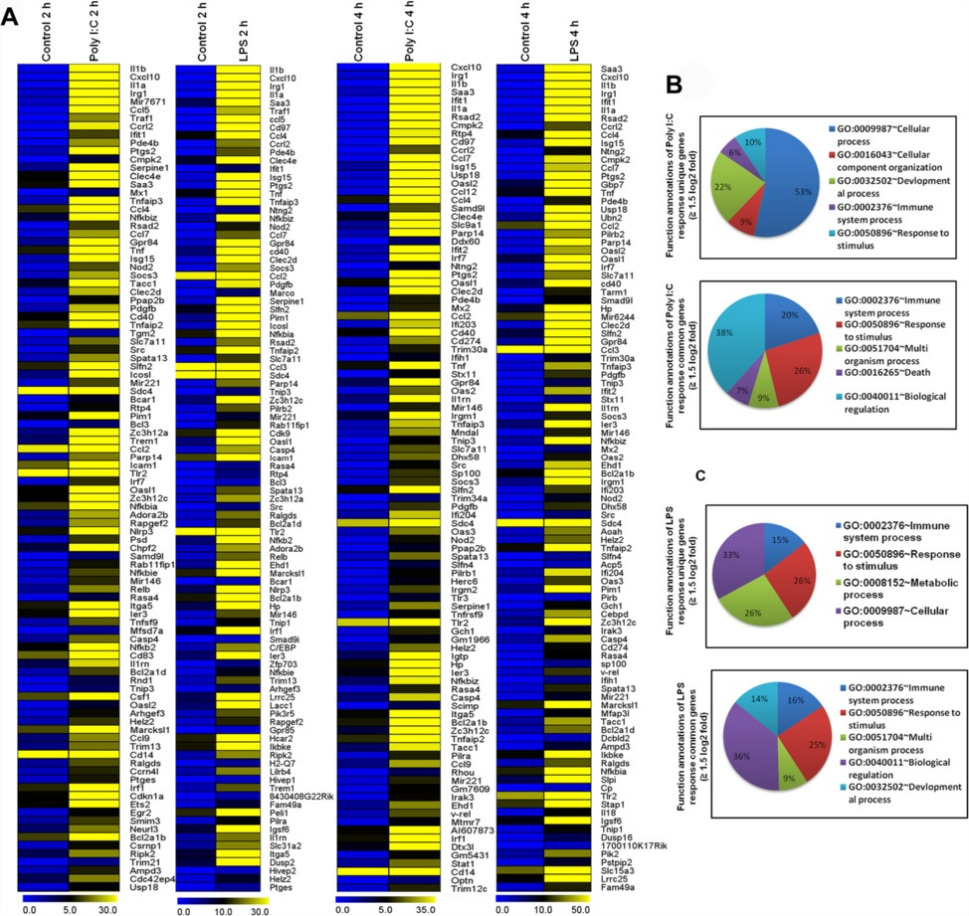
Differential gene expression and downstream effect analysis of genes overexpressed in microglia upon TLR3 and TLR4 stimulation at two different time points. **a** Heat map representing RNA-seq gene expression of top 150 up-regulated inflammatory genes at 2 and 4 h after Poly (I:C) and LPS stimulation in BV-2 microglia cells compared with controls. *P* <0.001, fold change ≥1.5 log_2_ for the significant determination of DEGs. Each row shows the relative expression level for a single gene, and each column shows the expression level of a single sample. Yellow represents genes with increased expression, and green represents genes with decreased expression. **b** and **c** Gene Ontology analysis of functional annotations (biological process) associated with Poly (I:C) and LPS-inducible up-regulated genes in BV-2 microglia in comparison with the control

The RNA-seq analysis revealed many genes are up-regulated in response to either Poly (I:C) or LPS stimulation, with many genes in common between the two TLR ligands. Importantly, we found that Poly (I:C) and LPS elicit the induction of a unique gene set in response to stimulation with this TLR ligand at both time points (Additional file 1). The genes were grouped into several categories based on their biological processes and molecular gene ontology functions, and heat maps were generated to aid the visualization of the gene expression pattern. The top 150 up-regulated inflammatory genes (*P* <0.001) at 2 and 4 h after Poly (I:C) and LPS stimulation in BV-2 microglia cells is depicted in Fig. 2a. Because the down-regulated genes were not associated with inflammation (data not shown), only the up-regulated genes were further studied. Next, we performed functional classification analyses of the up-regulated genes (*P* <0.001, and fold change ≥1.5 log_2_) using DAVID Informatics Resources through classification into GO categories (FDR 0.05) based on biological process (BP) and molecular function (MF) categories and on KEGG (Kyoto Encyclopedia of Genes and Genomes) pathways. The unique genes up-regulated in response to LPS stimulation were involved in stimulus responses and cellular processes, whereas the genes up-regulated in response to Poly (I:C) stimulation were involved in cellular processes. In contrast, both LPS- and Poly (I:C)-activated genes were involved in immune system processes, biological regulation and stimulus responses (Fig. 2b and c).

### Gene network analysis and canonical pathways modulated through TLR3- and TLR4-stimulated BV-2 microglial cells

**Fig. 3:**
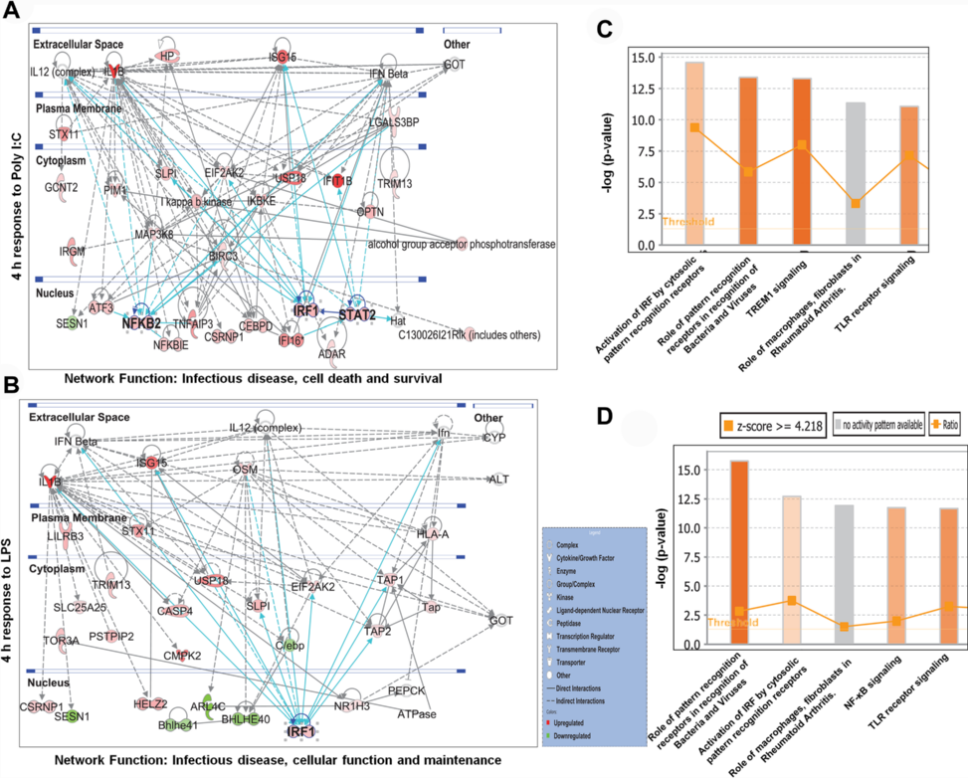
Top IPA-based network involved in infectious diseases and canonical pathway analyses at 4 h after TLR3 and TLR4 stimulation. **a**, **b** Ingenuity® Bioinformatics pathway analysis of gene networks displaying interactions between infectious disease-related genes that were differentially expressed at 4 h after Poly (I:C) and LPS stimulation. Genes in white circles were not in our DEG dataset but were inserted by IPA because these genes are connected to this network. The activity of molecules highly connected to this network, namely, NF-κB2, STAT2, and IRF1 (hubs), was assessed using the IPA molecule activity predictor. **c**, **d** The most highly represented canonical pathways for differentially expressed genes in BV-2 microglial cells after Poly (I:C) and LPS stimulation

The differentially expressed genes (DEGs) (*P* <0.001, and fold change ≥1.5 log_2_) were examined by IPA (IPA, Ingenuity Systems, http://www.ingenuity.com) to identify the canonical pathways by mapping these transcripts to the IPA program and to gain further insight into the molecular functions of these genes. Two metrics were used to recognize the most significant downstream effects of these overexpressed genes: the activation z-score and *P* value. A positive z-score implies increased functional activity. The *P* value, which is calculated using Fisher’s exact test, indicates the likelihood that the relationship between a set of genes in our dataset and a biological function is considerable. The top 5 canonical pathways for the differentially expressed genes at the two time points are displayed in Fig. 3c and d and Additional file 2 C and D. Almost all functions predicted to be activated in response to TLR3 and TLR4 stimulation (positive z-score) were related to the immune system; pattern recognition receptors play roles in bacteria and virus recognition NF-κB signaling, and TLR signaling. The up-regulation of these functions in response to TLR3 and TLR4 stimulation is interesting and supports the strength of our approach but is rather predictable. Next, we utilized IPA to explore the set of input genes to generate networks using the IPKB for interactions between identified DEGs. The IPA analysis revealed the top two networks of the differentially expressed genes established at either the 2 or 4 h time point after TLR3 and TLR4 stimulation in BV-2 cells. Networks 1 and 2 are illustrated in Fig. 3a and b and Additional file 2 A and B. Interestingly, the top networks involved in infectious diseases and inflammatory response were characterized by the activation of interferon regulatory factor 1 (IRF1), NF-κB2 and signal transducer and activator of transcription 2 (STAT2). The activation of IRF1, NF-κB2 and STAT2 has been reported in several models regarding inflammatory response [29, 30]. Of note, although IRF1 signals are essential for infectious diseases and for the inflammatory response of TLR4, IRF1, NF-κB2 and STAT2 were predicted to exhibit higher activation under TLR3 stimulation (Fig. 3a and b). The four networks depicted in Fig. 3a and b and Additional file 2 A and B show that several genes that are crucial to infectious diseases and inflammatory responses are expressed at higher levels in TLR3- and TLR4-stimulated BV-2 cells. Notably, this high expression was the case for Il-1ß, ISG15, and CASP4, which are crucial for infectious diseases [31–33].

### Differential induction of proinflammatory cytokines, chemokines and interferon response genes by TLR3 and TLR4 stimulation

**Fig. 4:**
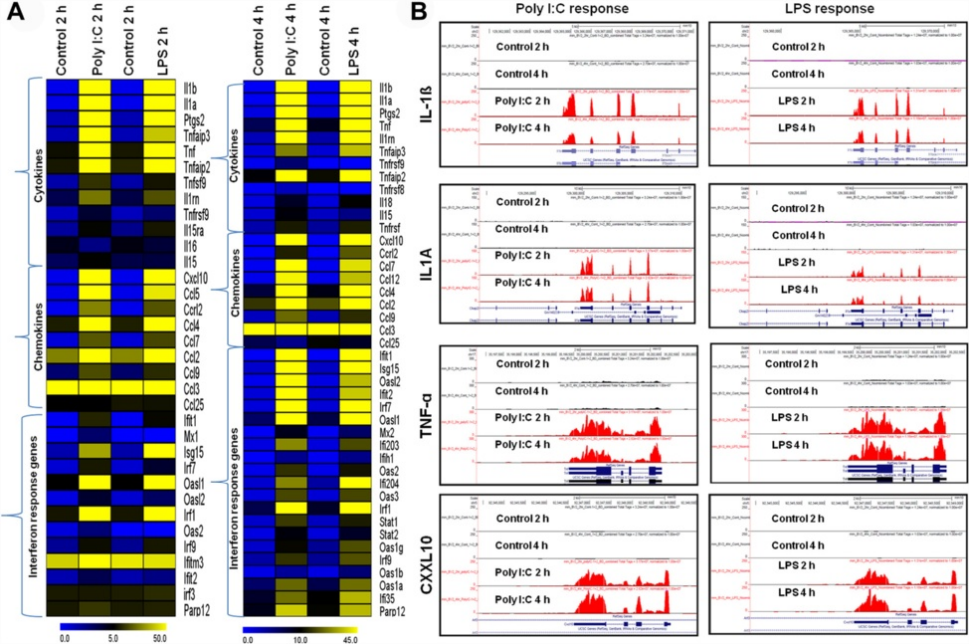
Inflammatory gene expression patterns in TLR3- and TLR4-stimulated BV-2 microglia cells. **a** Heat map representation depicting the expression of positive regulators of inflammatory genes (*P* <0.001; fold change ≥1.5 log_2_) at 2 and 4 h after Poly (I:C) and LPS stimulation compared with controls. **b** UCSC browser images representing normalized RNA-seq read densities after Poly (I:C) (left panel) and LPS (right panel) stimulation in BV-2 microglia cells compared with controls at the indicated times

The effects of Poly (I:C) and LPS treatments on cytokine, chemokine and interferon response gene expression were examined in BV-2 microglial cells to simplify the RNA-seq data analysis for the identification of TLR3 and TLR4-stimulated genes. Poly (I:C) induces pathways similar to those induced by LPS, leading to the production of proinflammatory cytokines such as IL-6, TNF-α and IFN-regulated genes (IRGs), as well as the chemokine CXCL10 in mouse cells [9]. As expected, our RNA-Seq analysis revealed that proinflammatory cytokines (IL-1ß and IL1A) were highly induced by Poly (I:C) and LPS stimulation in microglia, with Poly (I:C) being more potent than LPS. The reverse situation was true for TNF-α (Fig. 4a and b). Interestingly, unlike other proinflammatory cytokines, IL-6 was not induced in microglia. However, Poly (I:C) was described by another group to induce IL-6 mRNA in human dendritic cells (DCs) [34]. The chemokine displaying the highest level of up-regulation was the IRF3-dependent gene CXCL10. Interestingly, CXCL10-associated CXCR3-binding chemokines (CXCL9) and I-TAC (CXCL11) were not induced in Poly (I:C)- and LPS-stimulated BV-2 microglial cells. The GRO oncogenes (CXCL1 and CXCL3) observed a similar trend. Rantes (CCL5) and monocyte chemoattractant proteins (MCP) 1 and 3 (CCL2 and CCL7, respectively) were also up-regulated, with no clearly discernible pattern of induction by the two TLR ligands. CCR7, which is a DC antigen that is expressed in microglia in inflamed CNS tissues [35], stromal-derived factor 1 (SDF-1: CXCL12) and the recently discovered chemokine CCL25 were not induced. Next, we assessed the expression levels of several IRG genes with either the TLR3 agonist Poly (I:C) or the TLR4 agonist LPS. These IRGs included those involved in antiviral responses. These IRGs were among the most vastly induced genes in both the Poly (I:C) and LPS arrays. These highly induced genes included MX2, OAS2, OAS3, IFN-induced protein with tetratricopeptide (IFIT) family genes IFIT1 (p56) and IFIT2 (p54), IRF3-dependent genes ISG15 [32], IFI35, IFI203, and IFI204, with Poly (I:C) being more potent than LPS (Fig. 4a). Interestingly, we could not detect other IRGs including MX1, ISG20, IFN-induced transmembrane proteins IFITM1 and IFITM3, GBP1 and GBP2 [36] in Poly (I:C)- and LPS stimulated BV-2 microglial cells in this study. These data strongly suggest that both Poly (I:C) and LPS selectively induce proinflammatory cytokines, chemokines and interferon response genes in BV-2 microglial cells.

### Different TFs in multiple families of RNA-Seq data are activated by TLR3 and TLR4 stimulation in BV-2 microglial cells

**Fig. 5:**
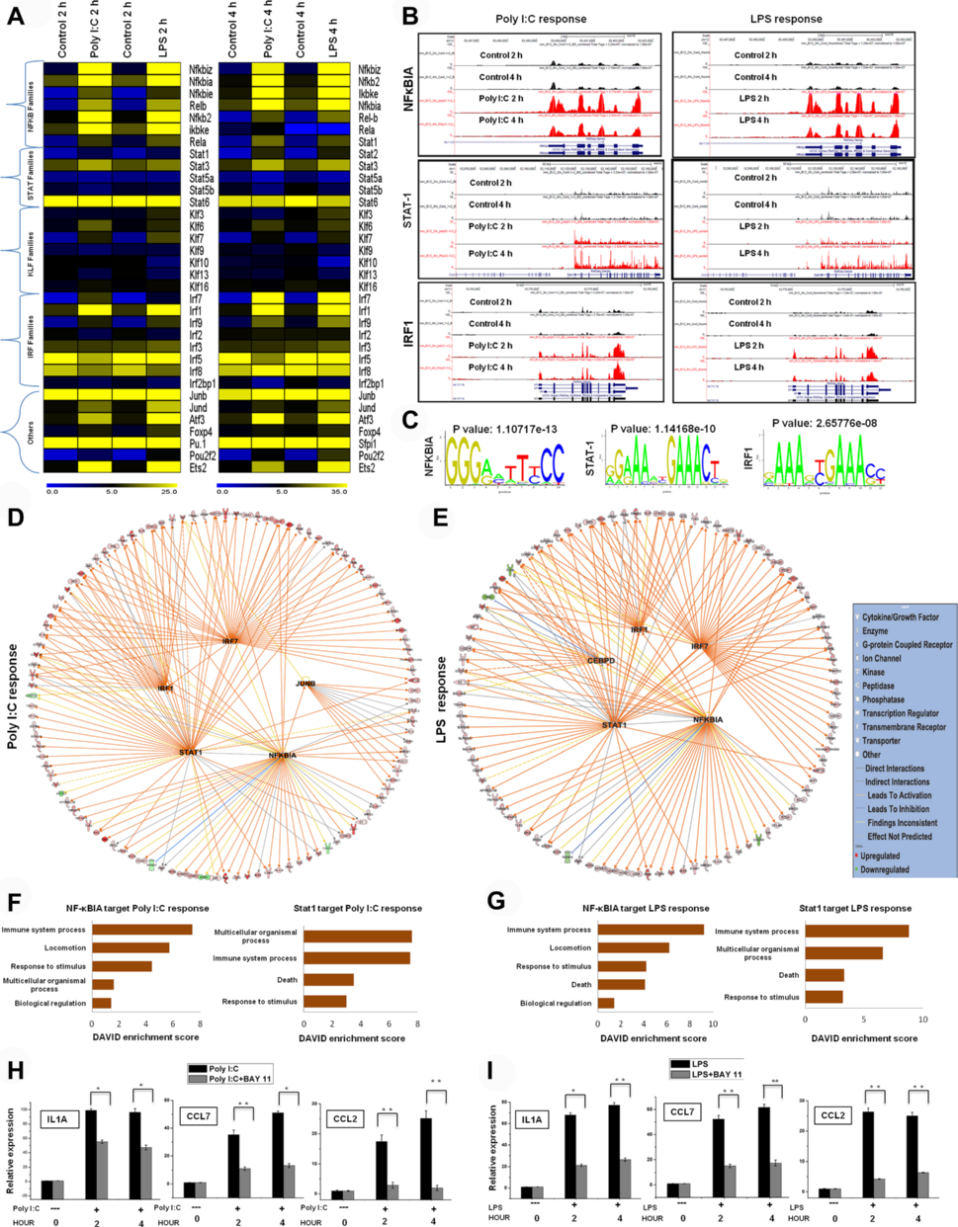
Transcriptomic analysis of selected TF families in BV-2 microglial cells. **a** Heat map represents differential expression of NF-κB, STAT, KLF, and IRF TF families, as well as other TF genes, (*P* <0.001) at 2 and 4 h after Poly (I:C) and LPS stimulation in BV-2 microglial cells. **b** UCSC browser images representing normalized RNA-seq read densities for TF expression after Poly (I:C) (left panel) and LPS (right panel) stimulation in BV-2 microglia cells compared with controls. **c** Patterns of transcription factor motif enrichments within the promoters of the genes in Poly (I:C)- and LPS-stimulated BV-2 microglia cells. **d**, **e** The activity of highly connected positive regulators of the inflammatory genes IRF1, IRF7, JUNB, NF-κBIA, STAT1, and CREPD led to the activation of this network, as assessed using the IPA molecule activity predictor in Poly (I:C)- and LPS-stimulated BV-2 microglia cells. **f**, **g** Results of the GO term analysis using DAVID on genes that were regulated by NF-κBIA and STAT1 in Poly (I:C) and LPS response BV-2 microglial cells respectively. (H, I) The IL1A, CCL7 and CCL2 genes were significantly down-regulated in NF-κB inhibitor Bay 11-7082 (10 μM)-treated BV-2 microglial cells at 2 h and 4 h under inflammatory conditions (Poly (I:C) 5 μg/ml and LPS 10 ng/mL). Gene expression was normalized to GAPDH transcript levels. *P <0.05 and **P <0.001 compared with the control. The data represent three independent experiments

Among the DEGs, multiple families of TFs were identified at 2 and 4 h after Poly (I:C) and LPS stimulation in BV-2 microglial cells (Fig. 5a and b). Poly (I:C) and LPS stimulation elicited a pronounced transcriptional response in BV-2 microglial cells at both time points. These TFs, including IRF, Kruppel-like factor (KLF), NF-κB and STAT, play important roles in neuroinflammatory diseases [29, 30]. The RNA-seq analysis revealed that STAT1 and IRF1 were highly induced by Poly (I:C) and LPS in microglia, with Poly (I:C) being more potent than LPS. The reverse situation was true for NF-κBIA and KLF7. Interestingly, another group found that Poly (I:C) was unable to activate NF-κB in DCs and macrophages (MØs) [37]. In contrast, our RNA-seq analysis revealed that NF-κB TFs were highly up-regulated in BV-2 microglial cells after Poly (I:C) stimulation. However, IRF2, IRF4, IRF6, IRF8, STAT6, KLF1, KLF2, KLF4, and KLF5 were unaffected after Poly (I:C) and LPS stimulation, suggesting that Poly (I:C)- and LPS-induced gene expression is highly selective in BV-2 microglial cells. In addition, the RNA-seq reads also revealed that JUNB, JUND, POU2F2, and ETS2 were particularly up-regulated by Poly (I:C) and LPS in microglia, with Poly (I:C) being more potent than LPS. The reverse situation was true for ATF3 and SFPI1 (PU.1) (Fig. 5a). Next, we conducted a TF motif analysis to assess Poly (I:C)- and LPS-induced gene expression in BV-2 microglial cells. We used the Pscan software tool [25] to perform the *in silico* computational analysis of over-represented *cis*-regulatory elements within the 5′-promoter regions of coordinately regulated genes. Applying this score to the promoters of the genes differentially expressed at 2 or 4 h (≥ 1.5 log_2_-fold) in response to Poly (I:C) and LPS stimulation revealed that the DNA sequences for NF-kB1, IRF1, and STAT1 were significantly enriched (Fig. 5c). In addition to TF motif analysis, we also used IPA software to identify the target genes that were directly or indirectly activated by identified TFs in response to TLR3 and TLR4 stimulation. Importantly, we found that the expression of the majority of cytokines and chemokines was directly regulated by the identified TFs, including IRF1, IRF7, STAT1, JUNB, CREPD, and NF-κBIA, whereas JUNB and CREPD target genes were unique in response to TLR3 and TLR4 stimulation, respectively (Fig. 5d and e and Tables 2 and 3). To further functionally classify the NF-κBIA and STAT1-regulated genes, it was functionally annotated using DAVID 6.7 software package. Interestingly, we observed strong enrichments of GO terms for the NF-κBIA and STAT1-regulated transcripts associated with the immune system processes, multicellular organism processes, locomotion and response to stimulus in Poly (I:C) and LPS response BV-2 microglial cells (Fig. 5f and g). To probe again whether NF-κB pathway inhibitor Bay 11-7082 [14–16] triggered deregulation of inflammatory genes, we measured the expressions of selected inflammatory genes upon exposure to Bay 11-7082 and we found that Bay 11-7082 significantly down-regulates the expression of NF-κB target genes in Poly (I:C) and LPS induced BV-2 microglial cells (Fig. 5h and i ). Taken together, these data strongly indicate that multiple families of transcription factors might be involved in the regulation of BV-2 microglial cell activation in response to TLR3 and TLR4 stimulation.

**Table 2:** Leads to activation of inflammatory genes by identified TFs in response to TLR3 stimulation

STAT1 predicted to be activated (45 genes)*p = 4.76 e-43*)	IRF7 predicted to be activated (45 genes) (*p = 1.33 e-43*)	NFKBIA predicted to be activated (35 genes) (*p = 5.31 e-30*)	IRF1 predicted to be activated (24 genes) (*p = 7.19 e-22*)	JUNB predicted to be activated (7 genes) (*p = 1.24 e-07*)
USP18, TRAFD1, TNF, TAP1, STAT2, SP110, SOCS3, SLFN5, SLFN2, SLFN13, SLFN12L, RSAD2, PTGS2, OASL, NEURL3, MX1/MX2, JAK2, ISG15, IRGM1, IRG1, IRF9, IRF7, IRF1, IL15, IGTP, IFIT2, IFIT1B, IFI35, IFI16, ICAM1, HERC6, EIF2AK2, CXCL10, CMPK2, CDKN1A, CD40, CD274, CD14, CCRL2, CCL2, CASP4, C3, APOBEC3B, CASP2, APOC2	USP18, UBA7, TRIM5, TRIM30A, TRIM21, TREX1, TAP1, STAT2, STAT1, SAMD9L, RTP4, RSAD2, PLSCR1, PHF11, PELI1, PARP14, PARP12, OASL2, OASL, OAS3, OAS2, OAS1, MX1/MX2, JAK2, ISG15, IRGM1, IRGM, IRF9, IRF1, IL15, IGTP, IFIT2, IFIT1B, IFIH1, IFI35, IFI16, HELZ2, DHX58, DAXX, CXCL10, CMPK2, CD40, CCRL2, CASP4, ADAR	TNFAIP3, TNFAIP2, SOCS3, SLFN2, SAA3, RCAN1, RASA3, PTGS2, PLAU, NFKBIE, NFKB2, JUNB, JUN, ISG15, IRG1, IRF1, IL1RN, IL1A, IL15, IFIT1B, IFI16, ICAM1, HMOX1, HLA-A, GCH1, CLU, CEBPD, CCL9, CCL7, CCL3L3, CCL2, CASP4, AMPD3, MXD4, CXCR4	TRIM21, TNF, TLR3, TAP1, STAT2, STAT1, RSAD2, PTGS2, PML, OAS1, JAK2, ISG15, IRF9, IRF7, IL1B, IL18, IL15, IFIT2, IFIH1, IFI35, EIF2AK2, CXCL10, CDKN1A, CD40, CASP2	TNFRSF8, SERPINE1, PLAUR, PLAU, HMOX1, BCL3, ATF3

**Table 3:** Leads to activation of inflammatory genes by identified TFs in response to TLR4 stimulation

IRF7 predicted to be activated (43 genes) (*p = 3.25 e-38*)	STAT1 predicted to be activated (36 genes) (*p = 3.02 e-32*)	NFKBIA predicted to be activated (35 genes) (*p = 5.88 e-25*)	IRF1 predicted to be activated (20 genes) (*p = 1.10 e-14*)	CEBPD predicted to be activated (11 genes) (*p = 1.22 e-10*)
USP18, XAF1, UBA7, TRIM5, TRIM30A, TRIM21, TREX1, TAP2, TAP1, STAT2, STAT1, SAP30, RSAD2, PHF11, PELI1, PARP14, PARP12, OASL2, OASL, OAS3. OAS2, OAS1, MX1/MX2, ISG15, IRGM1, IRGM, IRF9, IRF1, IRF7, IGTP, IFIT2, IFIT1B, IFIH1, IFI16, HELZ2, DHX58, DAXX, CXCL10, CMPK2, CCRL2, CASP4, ADAR, TLR8	USP18, TRAFD1, TNF, TAP1, STAT2, STAT1, SOCS3, SLFN5, SLFN2, SLFN13, SLFN12L, RSAD2, PTGS2, OASL, NEURL3, MX1/MX2, ISG15, IRGM1, IRG1, IRF9, IRF1, IRF7, IGTP, IFIT2, IFIT1B, IFI16, ICAM1, HERC6, EIF2AK2, CXCL10, CMPK2, CDKN1A, CD274, CD14, CCRL2, CASP4, C3, APOBEC3B,	TNFRSF1B, TNFAIP3, TNFAIP2, SOCS3, SLFN2, SAA3, RASA3, PTGS2, NFKBIE, NFKB2, NFKB1, JUNB, ISG15, IRG1, IRF1, IL1RN, IL1A, IFIT1B, IFI16, ICAM1, HMOX1, HLA-A, GCH1, CP, CFLAR, CEBPD, CEBPB, CCL9, CCL7, CCL2, CCL3L3, CASP4, BTG2, AMPD3, CXCR4	TRIM21, TNF, TLR3, TAP1, TAP2, STAT2, STAT1, RSAD2, PTGS2, OAS1, ISG15, IRF9, IRF7, IL1B, IL18, IFIT2, IFIH1, EIF2AK2, CXCL10, CDKN1A,	TNF, SAA3, PTGS2, PAX3, IKBKE, HP, CEBPB, CD14, CCL3, CCL2, C3

### Comparison of the transcriptional and post-transcriptional regulation by TLR3 and TLR4 stimulation in BV-2 microglial cells

**Fig. 6:**
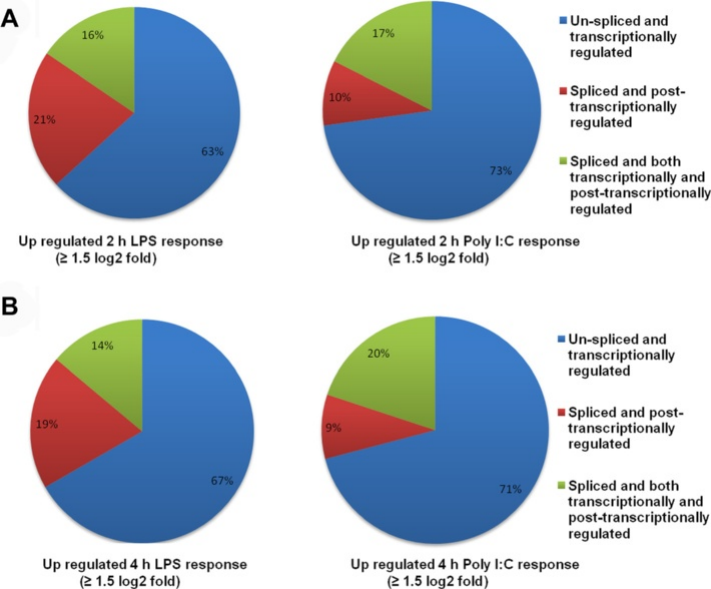
Transcriptional and post-transcriptional regulatory effects on overall transcript output in BV-2 microglia cells. **a**, **b** Pie charts showing the three categories of transcriptional and post-transcriptional regulation of genes at 2 h (upper panel) and 4 h (lower panel) after Poly (I:C) and LPS stimulation in BV-2 microglia cells

Transcriptionally regulated differentially expressed isoforms have different transcriptional start sites (TSSs), while post-transcriptionally regulated differentially expressed isoforms have the same TSSs [21]. The transcripts, isoforms and TSSs of the genes that were up-regulated (≥ 1.5 log_2_-fold) in Poly (I:C)- and LPS-stimulated BV-2 cells after 2 and 4 h were investigated in the present study. We defined the following three groups of genes (Fig. 6a and b): genes with one TSS and one isoform, which were classified as “un-spliced and transcriptionally regulated”; genes with one TSS and more than one isoform, which were classified as “spliced and post-transcriptionally regulated”; and genes with more than one TSS and more than one isoform, which were classified as “spliced and both transcriptionally and post-transcriptionally regulated”. Our RNA-seq analysis revealed that the highest percentages of genes that were both Poly (I:C)- and LPS-regulated were un-spliced and transcriptionally regulated. Importantly, this analysis also indicated that Poly (I:C) was more potent for spliced and both transcriptionally and post-transcriptionally regulated genes than LPS. The reverse situation was true for spliced and post-transcriptionally regulated genes (Fig. 6a and b).

### Different epigenetic regulators in multiple families of RNA-Seq data are activated by TLR3 and TLR4 stimulation in BV-2 microglial cells

**Fig. 7:**
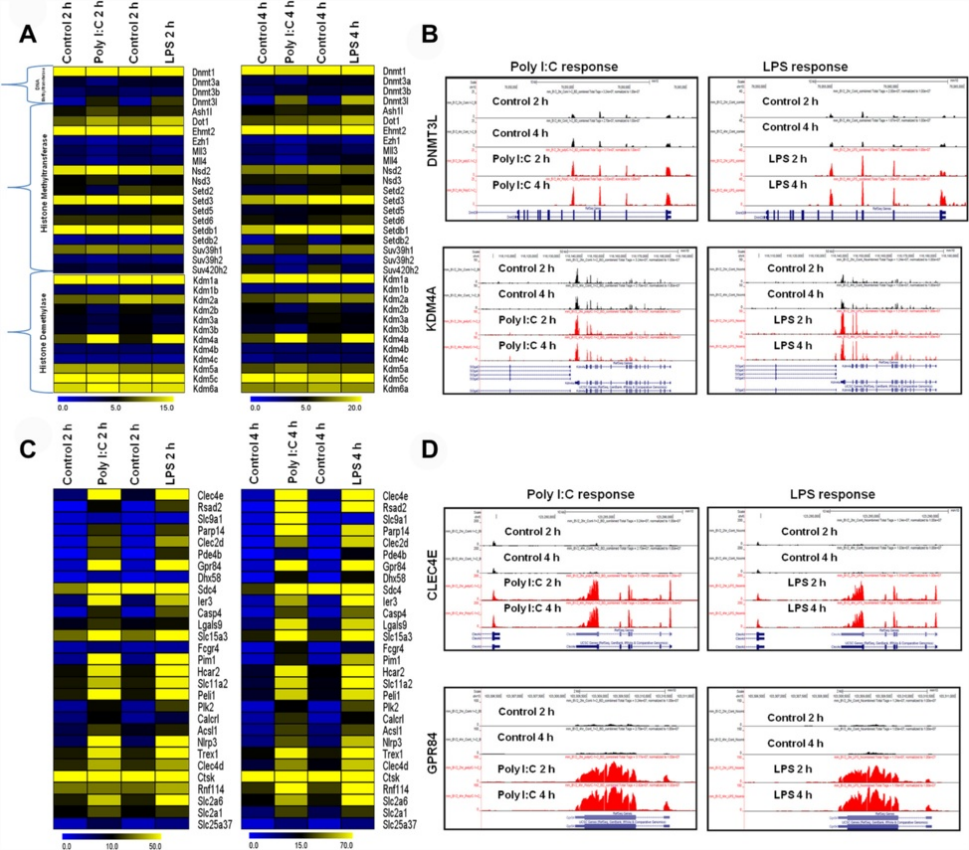
RNA-seq analysis reveals that TLR3 and TLR4 induce epigenetic regulators and novel inflammatory related genes. **a**, **c** Heat map representation depicting the expression of epigenetic regulators families and novel inflammatory related genes (*P* <0.001; fold change ≥1.5 log_2_) at 2 and 4 h after Poly (I:C) and LPS stimulation compared with controls. **b**, **d** UCSC browser images representing normalized RNA-seq read densities of epigenetic regulators and novel inflammatory related genes after Poly (I:C) and LPS stimulation in BV-2 microglia cells compared with controls at the indicated times

We also identified the most significant epigenetic regulators in multiple families that were altered in TLR ligand-stimulated BV-2 microglial cells; epigenetic regulation is defined as genetic control through factors other than the DNA sequence [38]. Studies of epigenetic regulation to potentiate innate immune responses have recently emerged [39]. Our RNA-seq analysis provides the first evidence that of multiple families of epigenetic regulators, only DNA methyltransferase (DNMT3L), histone methyltransferase (SETDB2), and histone demethylases (KDM4A) were particularly up-regulated by Poly (I:C) and LPS stimulation in microglia, with LPS being more potent than Poly (I:C). The reverse situation was true for histone methyltransferase SETDB2 (Fig. 7a and b), suggesting that DNMT3L, KDM4A, and SETDB2 might be involved in the regulation of BV-2 microglial cell activation.

### Identification of novel genes synergistically up-regulated by TLR3 and TLR4 stimulation in BV-2 microglial cells

Many studies have investigated the effects of TLR ligands, particularly TLR4, on cytokines, chemokines, and interferon response genes, as well as of different TF families on macrophages, which are important in inflammatory diseases. However, none of these studies has focused on novel genes that are also potentially involved in inflammatory diseases. To gain greater insight into mRNA expression studies, our RNA-seq analysis unexpectedly revealed approximately 29 previously unidentified genes that were particularly up-regulated by TLR ligands. Surprisingly, these 29 novel genes are frequently overexpressed not only in Poly (I:C)-induced microglial responses but also in LPS-induced microglial responses (Fig. 7c and d). Notably, the up-regulated genes that exhibited the significant links with inflammation included CLEC4E, RSAD2, PDE4B, GPR84, IER3, SLC15A3, CASP4, FCGR4, PELI1, PIK2, RNF114 and TREX, which are known to play important roles in inflammatory disorders [33, 40–42]. We observed that the RSAD2, FCGR4, and RNF114 genes displayed higher up-regulation upon Poly (I:C) stimulation than upon LPS stimulation. The reverse situation was true for PDE4B, IER3, SLC15A3, PIK2 and TREX.

### Confirmation of differentially expressed genes through qRT-PCR

**Fig. 8:**
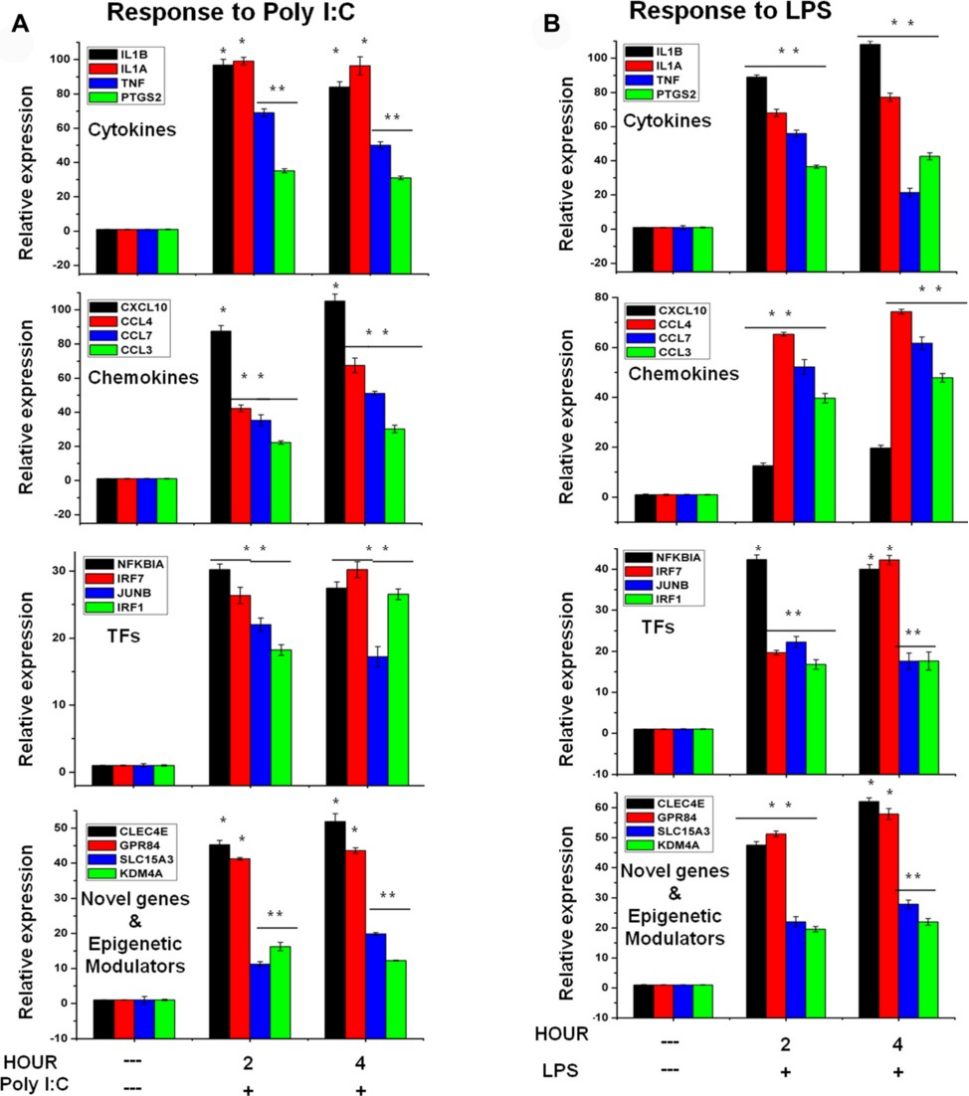
Confirmation of differentially expressed genes by quantitative reverse transcription-polymerase chain reaction. **a**, **b** IL-1ß, IL1A, TNF-α, PTGS2, CCL3, CCL4, CCL7, CXCL10, IRF1, IRF7, JUNB, NF-κBIA, CLEC4E, GPR84, SLC15A3 and KDM4A genes were significantly up-regulated in Poly (I:C)- and LPS-stimulated BV-2 microglia cells. Gene expression was normalized to the GAPDH transcript levels. *P <0.05 and **P <0.001 compared with control. The data represent three independent experiments

**Fig. 9:**
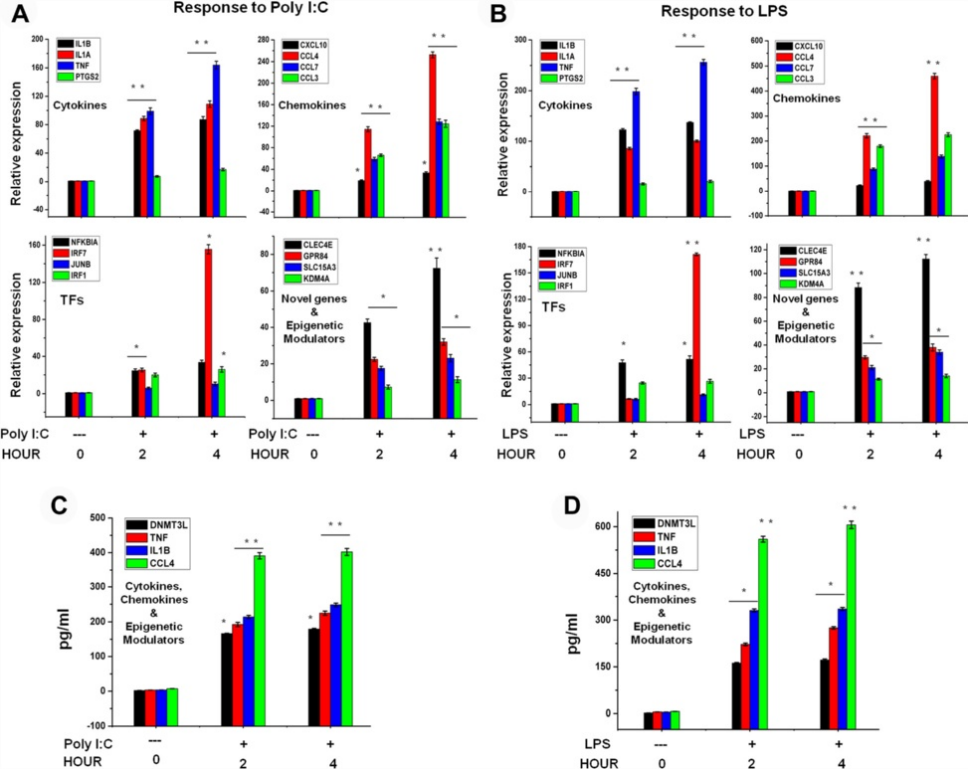
Confirmation of differentially expressed genes and release of pro-inflammatory mediators in primary microglial cells. **a** and **b** IL-1ß, IL1A, TNF-α, PTGS2, CCL3, CCL4, CCL7, CXCL10, IRF1, IRF7, JUNB, NF-κBIA, CLEC4E, GPR84, SLC15A3 and KDM4A genes were significantly up-regulated in Poly (I:C)- and LPS treated primary microglia cells. Gene expression was normalized to the GAPDH transcript levels. **c** and **d** Primary microglial cell culture supernatants of Poly (I:C)- and LPS treated cells were subjected to ELISA to detect the levels of pro-inflammatory cytokines/chemokines. Therefore, primary microglial cells were treated with Poly (I:C)- and LPS for 2 h and 4 h, followed by quantification of DNMT3L, TNF-α, IL-1ß and CCL4 levels. Values are given in pg/ml. Means and standard deviations of the mean of three independent experiments are shown (*P <0.05, **P <0.001, compared with control). The data represent three independent experiments

Many differentially regulated genes identified in the RNA-Seq analysis were subjected to validation through real-time qRT-PCR using GAPDH as a reference gene. The TLR3- and TLR4-affected genes were primarily selected for validation. To measure gene expression, mRNA was reverse transcribed into cDNA using Prime Script™ Reverse Transcriptase (Takara Bio Inc., Shiga, Japan); the qRT-PCR assays were repeated several times using at least 3 mRNA preparations from independent experiments. The results are expressed as fold changes relative to the control levels. Eighteen genes were selected for verification; the RNA-seq expression patterns were confirmed for sixteen genes (IL-1ß, IL1A, TNF-α, PTGS2, CCL3, CCL4, CCL7, CXCL10, IRF1, IRF7, JUNB, NF-κBIA, CLEC4E, GPR84, SLC15A3 and KDM4A; Fig. 8a and b and Tables 4 and 5), and the expression levels of two genes (KLF7 and IRAK3) were non-significant in the qRT-PCR analysis compared with the RNA-seq experiments (Tables 4 and 5). To confirm whether those genes were induced in primary microglial cells, we incubated primary microglial cells under inflammatory conditions Poly (I:C) (5 μg/ml) and (LPS 10 ng/mL), which induced inflammatory genes including IL-1ß, IL1A, TNF-α, PTGS2, CCL3, CCL4, CCL7, CXCL10, IRF1, IRF7, JUNB, NF-κBIA, CLEC4E, GPR84, SLC15A3 and KDM4A (Fig. 9a and b). Importantly, it should be noted that in primary microglial cells most of the inflammatory genes were induced stronger than BV-2 cell lines. In addition, we analyzed cytokines/chemokines in the supernatants of treated primary microglial cells with ELISAs. Compared to untreated cells DNMT3L, TNF-α, IL-1ß and CCL4 in the supernatants were increased in primary microglial cells following 2 h and 4 h Poly (I:C) (5 μg/ml) and (LPS 10 ng/mL) treatment (Fig. 9c and d).

**Table 4:** Comparison of fold change values from RNA-seq data and qRT-PCR in 2 h LPS and Poly I:C treated BV-2 microglia cells

RNA-seq fold change				qRT-PCR fold change	
Gene symbol	Gene accession ID	LPS_2h	Poly I:C_2h	LPS_2h	Poly I:C_2h
*Il-1ß*	NM_008361	93.82	96.13	89.02	96.72
*Il1A*	NM_010554	57.04	82.69	67.98	99.02
*TNF*	NM_001278601	51.12	45.94	55.94	69.01
*PTGS2*	NM_011198	32.41	51.93	36.5	35.09
*CXCL10*	NM_021274	34.74	88.57	12.53	87.45
*CCL4*	NM_013652	50.02	60.01	65.23	42.25
*CCL7*	NM_013654	17.41	44.98	52.23	35.26
*CCL3*	NM_011337	37.15	22.61	39.66	22.23
*NFκBIA*	NM_010907	38.57	33.60	42.36	30.21
*IRF7*	NM_001252600	21.59	34.28	19.68	26.35
*JUNB*	NM_008416	20.3	22.01	22.25	22.02
*IRF1*	NM_001159393	14.31	26.35	16.79	18.23
*CLEC4E*	NM_019948	53.91	53.29	47.52	45.25
*GPR84*	NM_030720	44.69	47.16	51.25	41.23
*SLC15A3*	NM_023044	18.0	16.23	22.03	11.23
*KDM4A*	NM_001161823	23.29	18.41	19.55	16.23
*KLF7*	NM_033563	4.89	3.85	1.04	0.909
*IRAK3*	NM_028679	4.97	4.25	0.77	0.89

**Table 5:** Comparison of fold change values from RNA-seq data and qRT-PCR in 4 h LPS and Poly (I:C) treated BV-2 microglia cells

RNA-seq fold change				qRT-PCR fold change	
Gene symbol	Gene accession ID	LPS_4h	Poly I:C_4h	LPS_4h	Poly I:C_4h
*Il1ß*	NM_008361	98.06	91.24	107.99	84.02
*Il1A*	NM_010554	66.01	82.29	77.23	96.32
*TNF*	NM_001278601	57.25	46.18	21.33	50.02
*PTGS2*	NM_011198	61.7	45.1	42.56	31.02
*CXCL10*	NM_021274	88.25	79.58	19.64	105.1
*CCL4*	NM_013652	69.71	61.29	74.25	67.52
*CCL7*	NM_013654	39.89	68.01	61.66	51.02
*CCL3*	NM_011337	45.34	30.07	47.85	30.21
*NFκBIA*	NM_010907	31.46	24.92	39.98	27.45
*IRF7*	NM_001252600	51.23	54.66	42.25	30.21
*JUNB*	NM_008416	18.46	16.16	17.58	17.23
*IRF1*	NM_001159393	17.02	31.08	17.61	26.53
*CLEC4E*	NM_019948	57.85	57.51	62.03	51.89
*GPR84*	NM_030720	45.93	45.79	57.89	43.58
*SLC15A3*	NM_023044	30.12	27.72	27.89	19.86
*KDM4A*	NM_001161823	17.16	11.20	22.00	12.23
*KLF7*	NM_033563	4.22	3.24	1.05	0.896
*IRAK3*	NM_028679	6.99	7.13	1.22	1.102

## Discussion

Microglial cells become rapidly activated through interactions with pathogens, and their persistent activation is associated with the production and secretion of various pro-inflammatory genes, cytokines and chemokines, which may initiate or amplify neurodegenerative diseases [5]. Previous studies have reported that LPS stimulation induces the gene expression of TNF-α, IL-1β, IL-6, iNOS and PTGS-2 and the production of NO and PGE2 in primary and BV-2 microglial cell cultures [6]. In contrast, Poly (I:C) activates antiviral and inflammatory genes independent of the adaptor protein MyD88, which is required for all other TLRs [3]. However, none of these studies addressed the effects of TLR ligands Poly (I:C) and LPS; thus, their responses were compared using RNA-seq analysis in microglial cells in the present study. This study provides the most comprehensive analysis thus far because, compared to microarrays, this technique can provide unbiased profiles, can be extremely accurate, and can identify novel transcribed regions if a sufficient level of coverage is obtained [43, 44]. Furthermore, these technologies facilitate the determination of the difference between the expression of alternative mature mRNAs from the same precursor and the identification of the differential expression of mRNA isoforms [43–45]. Validation techniques such as qRT-PCR [46] have corroborated the accuracy of RNA-seq.

The RNA-seq analysis revealed that cytokines/chemokines, antiviral genes, and IRGs associated with inflammation were significantly up-regulated in response to TLR3- and TLR4-induced microglial activation. Both the extent of the fold change and the number of genes were significantly modulated. The activation of TNF-α, IL-1β, IL-1A, PTGS-2, CXCL10 (IP-10), CCL7, CCL12, CCL4, and CCL2, all occurred similarly, with minor variations (Fig. 4a and b). IL-1 is the most widely studied pro-inflammatory gene; the extensively characterized forms of IL-1 are IL-1A and IL-1β. IL-1A and IL-1β play a crucial role in the development of AD and PD, the pathogenic hallmark of which is CNS inflammation. Following CNS damage, IL-1 is rapidly released from activated microglia, and an elevated level of the IL-1 cytokine is an important hallmark of neuroinflammation [26]. PTGS-2 is the key enzyme responsible for brain inflammation, and increased PTGS-2 expression contributes to neurodegeneration [47]. Chemokines are key regulators of inflammation, and the excessive production of these molecules has been associated with disease progression and severe inflammation pathologies, including MS. Conductier *et. al.(2010),* reported that CCL2 plays a crucial role in neuroinflammatory diseases and is considered a target in the treatment of neuroinflammatory disorders [48]. CCL2 and CCL7 are highly expressed in microglia, astrocytes and other inflammatory cells during MS [49]. CCL12 also plays an inflammatory role because the levels of this chemokine are up-regulated in both microglia and astrocytes when stimulated with the proinflammatory cytokine IL-17 [50]. The expression of CXCL10 is observed during infectious and inflammatory diseases, playing a crucial role in T-cell-mediated inflammation in the CNS. In addition, CXCL10 plays a role in inflammatory demyelinating diseases, such as MS, through the destruction of the myelin sheath or neurons by facilitating leukocyte trafficking in the brain [51]. These patterns are consistent with our studies showing the consequences of TLR3 and TLR4 stimulation with respect to the induction of cytokines/chemokines (Fig. 4a and b).

Various signaling pathways, including phosphoinositide 3-kinase, Jun N-terminal kinase (JNK), p38, NF-κB, extracellular signal-related kinase, and IRF3, have been activated by TLR3/TLR4, leading to the induction of numerous target genes involved in antiviral immunity, including IFN-ß and IP-10 production [9]. Our RNA-seq data revealed that the induction of several genes involved in antiviral immunity and signaling was more potent in TLR3-stimulated BV-2 microglial cells than in TLR4-stimulated BV-2 microglial cells and included IFIT1, ISG15, IFIT2, IFI203, IFI204, IRF1, IRF7, IRF9, STAT1, and STAT2 (Fig. 4a). These results were rather anticipated because TLR3 is unique, recruiting only TRIF and not MyD88. Unexpectedly, we were unable to identify IRF3 and its target gene IFN-ß, but not IP-10, in TLR3/TLR4-stimulated BV-2 microglial cells. Our data show consequences similar to that in a previously published report regarding the induction of IRF3 in DCs and MØs [37]. However, because no IRF3 activation could be observed in BV-2 microglia cells, the mechanism by which the production of IP-10 and IRGs is regulated in these cell types remains unclear. This mechanism is the subject of ongoing investigations. Furthermore, to evaluate the expressions of IRF3, IFN-ß, and IP-10 (CXCL10) on TLR3- and TLR4-induced primary microglial cells we measured the expressions of IRF3, IFN-ß, and IP-10 for 2 h and 4 h time points. Interestingly, we found that IFN-ß, and IP-10 were significantly up-regulated in primary microglial cells (Additional file 3 A and B and Fig. 9a and b). Although, primary microglial cells with these factors did not induce the expression of IRF-3 (Additional file 3 A and B). Nevertheless further studies are warranted to assess genome wide transcriptomic analysis on TLR3-and TLR4-stimulated primary microglial cells. Importantly, we first identified IRF1, IRF7, and IRF9 as significantly up-regulated in response to TLR3- and TLR4-stimulated microglia (Fig. 5a). A previous study reported that IRF7 could be a master regulator of type-I IFN-mediated responses [52]. In addition, IRF1 has been suggested to be a master transcription factor contributing to IRGs [53]. Thus, exploring whether interferon response gene induction mechanisms not relying on IRF3 may exist in cells other than microglia or macrophages would be interesting. Therefore, IRF7, IRF1, and IRF9 most likely do not act in place of IRF3 in BV-2 microglia cells in response to TLR3 and TLR4 stimulation. This possibility is an exciting area that we are keenly pursuing further.

In addition to IRF TFs, we identified NF-κB and STAT, as well as additional TFs (PU.1, KLF7, JUNB, ATF3, and FOXP4) whose roles in microglia activation have not yet been well established (Fig. 5a and b). We observed the significant up-regulation of NF-κB transcription factor, which was induced by Poly (I:C) and LPS stimulation in microglia, with LPS being more potent than Poly (I:C). Similar to our findings, Reimer *et. al*. (2008) reported that Poly (I:C) and LPS induce the activation of NF-κB, followed by the release of TNF-α [9]. JUNB also plays an important role in controlling activity-dependent gene programs that are critical for nervous system function [54]. However, IRF2, IRF4, IRF6, IRF8, STAT6, KLF1, KLF2, KLF4, and KLF5 were unaffected by Poly (I:C) and LPS stimulation, suggesting the highly selective induction of TFs through TLR3 and TLR4 stimulation in BV-2 microglial cells. To delineate conserved transcription factor-binding motifs further, we performed TF motif analysis on TLR3- and TLR4-stimulated genes in BV-2 microglial cells. The core promoters of co-expressed genes (typically, regulatory regions within -1000 to +50 bp relative to the TSS) can be evaluated for overrepresented *cis*-regulatory elements after partitioning into suitable modules [55]. Of the two ranges available in Pscan software that are closest to this region of interest (-950 to 50 and -1000 to 0), the -950 to +50 bp range was selected for the analyses. The promoters of differentially expressed genes revealed the enrichment of DNA sequences not only for NF-κB transcription factors but also for IRF1 and STAT1, as shown in Fig. 5c. These analyses converged the first insights into 3 TF binding motifs that may be involved in regulating subset-specific genes in Poly (I:C)- and LPS-stimulated BV-2 microglial cells. Next, we used IPA software to identify the target genes that were directly or indirectly activated by the identified TFs in response to TLR3 and TLR4 stimulation. Importantly, we found that the majority of the cytokines, chemokines whose expression is directly regulated by the identified TFs, including IRF1, IRF7, STAT1, JUNB, CREPD, and NF-κBIA, where JUNB and CREPD target genes were unique in response to TLR3 and TLR4, respectively (Fig. 5d and e and Tables 2 and 3).

We performed the first functional analysis of the transcripts, isoforms and TSSs to gain greater insight into the functional categories for Poly (I:C) and LPS-stimulated inflammatory genes in BV-2 microglial cells. Most of the differentially expressed genes in microglial following Poly (I:C) and LPS stimulation were expressed as several isoforms subjected to transcriptional/post-transcriptional regulation and/or to differential promoter usage. We classified these genes into three primary groups (genes with one isoform and one TSS; genes with more than one isoform and one TSS; and genes with more than one isoform and more than one TSS) (Fig. 6a and b). The first two groups included genes crucial for the innate immune response, which might be under stronger selection to prevent the emergence of new isoforms and/or post-transcriptional regulation. In addition, some genes belonged to the third group [56], suggesting that these genes could be subjected to positive selection by transcriptional and post-transcriptional regulation in Poly (I:C)- and LPS-stimulated BV-2 microglial cells. Interestingly, we found that spliced and both transcriptional and post-transcriptional regulated genes were greater in Poly (I:C)-stimulated BV-2 microglia than in LPS-stimulated BV-2 microglia after unspliced and transcriptionally regulated genes. The reverse situation was true for LPS-stimulated genes. However, further targeted studies are required to validate this regulation and to establish the potential effects of these genes during TLR3 and TLR4 stimulation in microglia.

Another interesting finding is the increased expression of epigenetic regulators, which involves chemical modifications of DNA cytosine residues and DNA-bound histone proteins without alterations in the DNA sequence; epigenetic regulation is promising as one of the major factors regulating gene expression in response to environmental stimuli [38]. Recent studies have demonstrated that histone demethylase (KDM6B) and histone deacetylases (HDAC1, HDAC2, HDAC3, and HDAC7) potentially regulate proinflammatory gene expression in macrophages [39, 57, 58]. Recently, we showed that the histone demethylase KDM4A was significantly expressed in neuroectodermal stem cells and might play a role in tumorigenic development [19]. Interestingly, among different epigenetic regulators, only histone demethylase KDM4A and DNA methyltransferase DNMT3L were strikingly differentially expressed in Poly (I:C)- and LPS-stimulated BV-2 microglial cells, with LPS being more potent than Poly (I:C), as determined by the RNA-Seq data in the present study (Fig. 7a and b). The mechanism by which KDM4A and DNMT3L become activated following TLR3 or TLR4 receptor stimulation remains unknown; determining how these epigenetic regulators, along with modified TFs, can regulate distinct set of inflammatory genes in microglial cells would be intriguing. We posit that the role of these genes in inflammatory diseases should now be assessed in animal models involving TLR-specific gene deletion or overexpression. However, histone demethylase (KDM6B) and histone deacetylases (HDAC1, HDAC2, HDAC3, and HDAC7), were not expressed in either Poly (I:C)- or LPS-stimulated BV-2 microglial cells.

One of the most striking features is that our RNA-seq analysis was the first to identify several notable differences in the pattern of previously unidentified genes activation induced by these distinct TLR ligands in BV-2 microglial cells. In particular, this technology allowed us to identify over 25 direct TLR3- or TLR4-stimulated genes in these cells. The direct TLR3- or TLR4-stimulated genes known to be important for activity-regulated processes in microglia include the macrophage-inducible C-type lectin (CLEC4E), which regulates immune responses to pathogens [40]; the secreted endo-lysosomal peptide transporter SLC15A3, which is highly expressed by bone marrow-derived dendritic cells after LPS stimulation and which plays a key role in regulating innate immune responses [42]; orphan G protein-coupled receptor 84 (GPR84), which is highly expressed in leukocytes, monocytes, and macrophages upon activation by LPS and which plays a critical role in immunological regulation [41]; and caspase-4, which plays an important role in inflammatory diseases [33]; (Fig. 7c and d). This finding indicates that these TLR3 or TLR4 targets also mediate the effects of the inflammatory response in mouse microglia. Furthermore, we confirmed the expression of key inflammation- and immunity-related genes as well as cytokines/chemokines in the supernatants were significantly induced in Poly (I:C) and LPS treated primary microglial cells including IL-1ß, IL1A, TNF-α, PTGS2, CCL3, CCL4, CCL7, CXCL10, IRF1, IRF7, JUNB, NF-κBIA, CLEC4E, GPR84, SLC15A3 and KDM4A (Fig. 9a, b, c and d). Future studies determining how these TLR3 or TLR4 target genes are expressed during the immune response in mouse microglia will be useful. This mechanism is the subject of ongoing investigations.

Overall, our RNA-seq data provide novel insights into the transcriptional landscape TLR3 and TLR4 ligands mimic certain aspects of viral infection by triggering key molecular events such as a rapid innate immune response during the early course of the inflammatory response. Regardless of certain boundaries in exactitude, TLR3- and TLR4-stimulated inflammatory gene expression profiles, TF and epigenetic regulator clusters, and microglial *cis*-regulatory elements predictions offer valuable information for future studies, such as potential gene targets for chromatin immunoprecipitation (ChIP)-seq assays. In the future, this model can be extended to include data from other high-dimensional surveys, such as microRNA, ChIP-seq, and proteomics, providing further insight into global gene regulation in TLR3- and TLR4-stimulated microglial cells.

## Conclusion

In summary, using RNA-seq, our study is the first to identify family-wide DEGs of most known and unknown immune genes that characterize the microglial response to two TLR stimuli, Poly (I:C) and LPS and to demonstrate both common and unique patterns. In addition, our data argue that, while both TLR3 and TLR4 have been evolutionarily selected to induce antiviral gene expression, TLR3 seems to be even more specialized than TLR4 to initiate antiviral responses and that the reverse situation was true for the master TF NF-κB. Furthermore, we examined epigenetic regulators, different TFs, and TF-regulated genes, as well as the transcriptional and post-transcriptional regulation of genes based on their isoforms, TSSs and differential promoters in two TLR-stimulated BV-2 microglial cells. These unprecedented results together indicate that both known and unknown DEGs, TFs, and epigenetic regulators identified in the present study may provide new insight regarding the innate immunity to TLR3 and TLR4 in BV-2 microglial cells and may enhance our knowledge of neuroinflammation.

### Availability of supporting data

The acquired data were deposited in the NCBI sequence read archive (http://www.ncbi.nlm.nih.gov/Traces/sra/sra.cgi?view=run_browser) under dataset accession no. SRR1554368, SRR1598823, SRR1554452, SRR1554456, SRR1554369, SRR1598824, SRR1554453, SRR1554457, SRR1692678, SRR1692736, SRR1692771, and SRR1692789.

## Additional files

Additional file 1:
**Identification of unique and shared genes.** Venn diagram displaying the number of unique or shared up-regulated genes at 2 h (left panel) and 4 h (right panel) after Poly (I:C) and LPS stimulation (*P* <0.001; fold change ≥1.5 log_2_) in BV-2 microglia cells. (TIFF 485 kb) Additional file 2:
**Top IPA-based network involved in infectious diseases and canonical pathway analyses at 2 h after TLR3 and TLR4 stimulation.** (A, B) Ingenuity^®^ Bioinformatics pathway analysis of gene network displays interactions between infectious disease-related genes that were differentially expressed at 2 h after Poly (I:C) and LPS stimulation. Genes in white circles were not in our DEG dataset but were inserted by IPA because these genes are connected to this network. The activity of molecules highly connected to this network, namely, IRF1, IRF7, and NF-κB (hubs), was assessed using the IPA molecule activity predictor. (C, D) The most highly represented canonical pathways for differentially expressed genes in BV-2 microglial cells after Poly (I:C) and LPS stimulation. Additional file 3:
**Effect of Poly (I:C) and LPS on the expressions of IFN-ß and IRF3 in microglial cells.** (A and B) Quantitative real-time reverse transcriptase-PCR analysis of IFN-ß and IRF3 gene expression in BV-2 and primary microglial cells stimulated with Poly (I:C) and LPS. Only the expression of IFN-ß was up-regulated in primary microglial cells compared with untreated cells (***P* <0.001, compared with control) at the indicated times. Gene expression was normalized to GAPDH transcript levels. The data represent three independent experiments. The values are shown as the means ± SD of triplicate wells. (C and D) UCSC browser images representing normalized RNA-seq read densities of IFN-ß after Poly (I:C) and LPS stimulation in BV-2 microglia cells compared with controls.
