# Supplementary figures and images for: Transcriptome sequencing of microglial cells stimulated with TLR3 and TLR4 ligands
Source: BMC Genomics. 2015 Jul 10;16(1):517. doi: 10.1186/s12864-015-1728-5 (PMC4497376; doi:10.1186/s12864-015-1728-5)

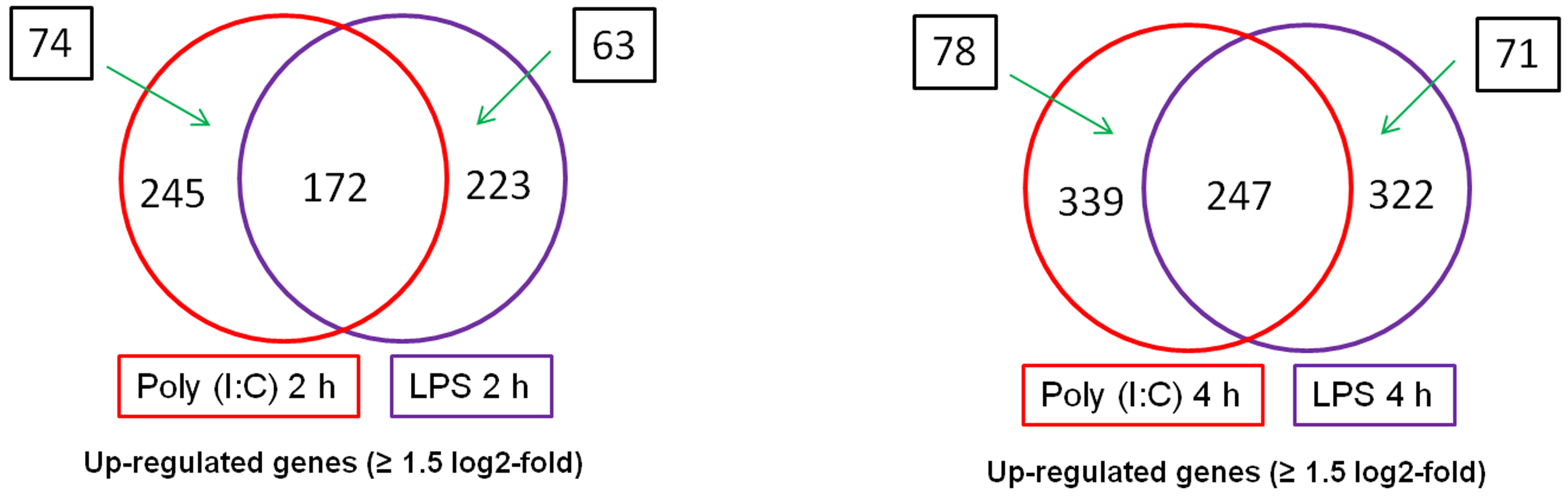

Supplement: Additional file 1: — Identification of unique and shared genes. Venn diagram displaying the number of unique or shared up-regulated genes at 2 h (left panel) and 4 h (right panel) after Poly (I:C) and LPS stimulation (P <0.001; fold change ≥1.5 log2) in BV-2 microglia cells. (TIFF 485 kb) [file 12864_2015_1728_MOESM1_ESM.tiff]

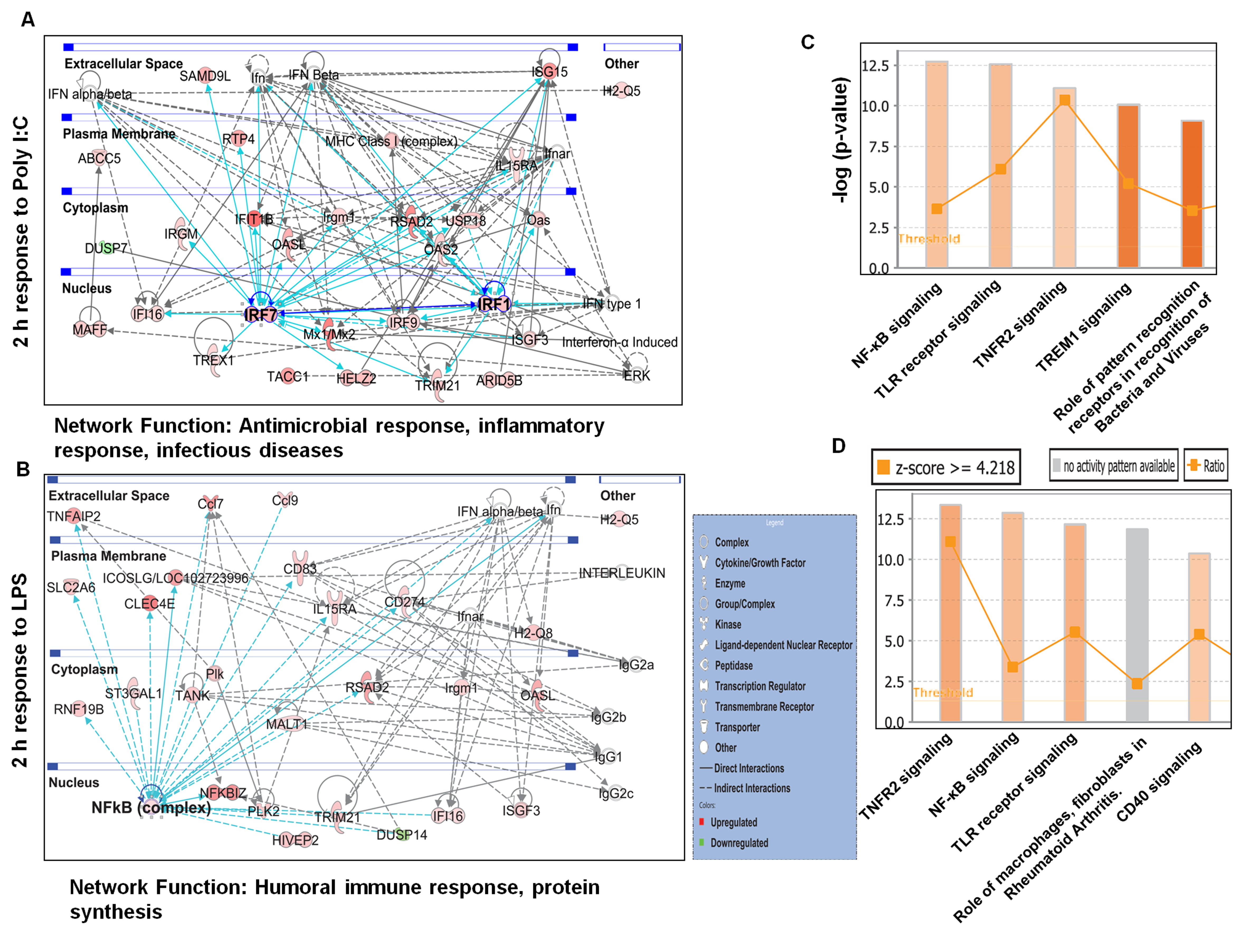

Supplement: Additional file 2: — Top IPA-based network involved in infectious diseases and canonical pathway analyses at 2 h after TLR3 and TLR4 stimulation. (A, B) Ingenuity® Bioinformatics pathway analysis of gene network displays interactions between infectious disease-related genes that were differentially expressed at 2 h after Poly (I:C) and LPS stimulation. Genes in white circles were not in our DEG dataset but were inserted by IPA because these genes are connected to this network. The activity of molecules highly connected to this network, namely, IRF1, IRF7, and NF-κB (hubs), was assessed using the IPA molecule activity predictor. (C, D) The most highly represented canonical pathways for differentially expressed genes in BV-2 microglial cells after Poly (I:C) and LPS stimulation. [file 12864_2015_1728_MOESM2_ESM.tiff]

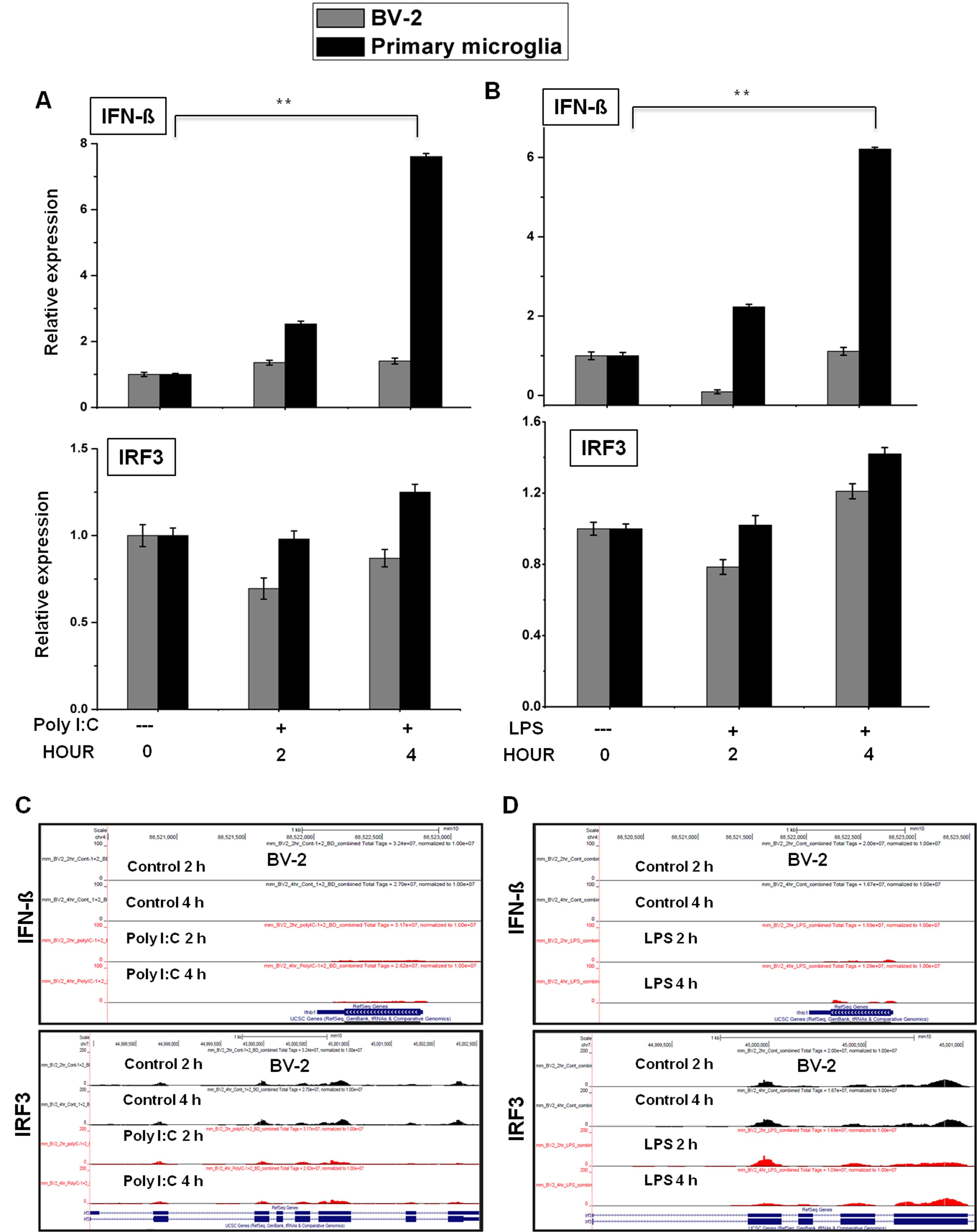

Supplement: Additional file 3: — Effect of Poly (I:C) and LPS on the expressions of IFN-ß and IRF3 in microglial cells. (A and B) Quantitative real-time reverse transcriptase-PCR analysis of IFN-ß and IRF3 gene expression in BV-2 and primary microglial cells stimulated with Poly (I:C) and LPS. Only the expression of IFN-ß was up-regulated in primary microglial cells compared with untreated cells (**P <0.001, compared with control) at the indicated times. Gene expression was normalized to GAPDH transcript levels. The data represent three independent experiments. The values are shown as the means ± SD of triplicate wells. (C and D) UCSC browser images representing normalized RNA-seq read densities of IFN-ß after Poly (I:C) and LPS stimulation in BV-2 microglia cells compared with controls. [file 12864_2015_1728_MOESM3_ESM.tiff]
